# Complete Genome Sequence of *Corynebacterium pseudotuberculosis* Strain PA01, Isolated from Sheep in Pará, Brazil

**DOI:** 10.1128/genomeA.01664-15

**Published:** 2016-01-28

**Authors:** Jorianne T. C. Alves, Adonney A. O. Veras, Ana Lídia Q. Cavalcante, Pablo H. C. G. de Sá, Larissa M. Dias, Luis C. Guimarães, Ezequiel Morais, André G. M. Silva, Vasco Azevedo, Rommel T. J. Ramos, Artur Silva, Adriana R. Carneiro

**Affiliations:** a Federal University of Pará, Center of Genomics and System Biology, Laboratory of Genomic and Bioinformatics, Belém, Pará, Brazil; b Federal University of Pará, Campus of Castanhal, Pará, Brazil; c Institute of Biological Sciences, Federal University of Minas Gerais, Belo Horizonte, Minas Gerais, Brazil

## Abstract

*Corynebacterium pseudotuberculosis* is the etiological agent of caseous lymphadenitis disease. In this work, we present the first complete genome sequence of *Corynebacterium pseudotuberculosis* strain PA01, isolated in northern Brazil from an infected sheep. The genome length is 2,337,920 bp, and 2,003 coding sequences (CDS), 12 rRNAs, and 49 tRNAs were predicted.

## GENOME ANNOUNCEMENT

*Corynebacterium pseudotuberculosis* is a facultative intracellular Gram-positive bacterium that belongs to the CMNR group (*Corynebacterium*, *Mycobacterium*, *Nocardia*, and *Rhodococcus*) (1), which causes caseous lymphadenitis (CLA), an infectious disease that affects small ruminants, mainly sheep and goats, and is characterized by the formation of abscesses in the superficial lymph nodes and subcutaneous tissues (1, 2).

This disease causes economic loss due to the progressive reduction in weight gain, depreciated wool and skin, reduced milk production, and eventually death caused by toxemia of the infected animals. It impacts sheep and goat farming around the globe, especially in the United States, Australia, South Africa, and Brazil (1, 3, 4). In Brazil, the agribusiness of goat and sheep has increased, especially in the northeast (http://www.agricultura.gov.br) and, consequently, there is a high prevalence of CLA disease in the states of Bahia (5), Pernambuco (6), and Rio Grande do Sul (7).

However, despite the bacteria epidemiology, there are no reports of its isolation in northern Brazil. *C. pseudotuberculosis* strain PA01 was isolated from the lymph nodes of a sheep in Pará, Brazil. Biochemical identification was performed using the API Coryne kit (bioMérieux, USA) and the strain was characterized as biovar *ovis*. Though it is nitrate reductase negative (8), molecular biology confirmation was obtained by a PCR multiplex with *rpoB*, *16S*, and *pld* genes (9). The genome was sequenced by the Ion Torrent PGM platform using a fragment library, which produced 1,894,790 reads. After sequencing, the reads were evaluated for quality using FastQC (http://www.bioinformatics.babraham.ac.uk/projects/fastqc/), and filtered and trimmed with average Phred quality scores equal to or greater than 20 by the FastX toolkit (http://hannonlab.cshl.edu/fastx_toolkit/), followed by assembly using Mira (10), which generated 22 contigs. The Lasergene 11 Core Suite with the SeqMan Proo tool was used to reduce the number of contigs to 5. The scaffold was obtained using Mauve (11) with *C. pseudotuberculosis* strain FRC41 (NC_014329) as the reference genome. Artemis software was utilized to edit and fill gaps (12). Automatic annotation was performed using Rapid Annotation using Subsystem Technology (RAST) (13). The prediction of rRNAs and tRNAs were performed using RNAmmer (14) and tRNAscan-SE (15), respectively. The identification of protein domains and families was performed by InterproScan (16). All coding sequences (CDS) were manually curated using Artemis (12), BLASTp (http://blast.ncbi.nlm.nih.gov/), and the UniProt (http://www.uniprot.org) database. The identification and validation of the pseudogene was done using CLC Genomics Workbench (http://www.clcbio.com/).

The *C. pseudotuberculosis* strain PA01 genome consists of a circular chromosome of 2,337,920 bp, with 52.18% G+C content, 2,003 CDS, 12 rRNA operons, 49 tRNAs, and 17 pseudogenes predicted.

### Nucleotide sequence accession number.

This genome project has been deposited in GenBank under the accession number CP013327.
