# Erratum for Alves et al., Complete Genome Sequence of *Corynebacterium pseudotuberculosis* Strain PA01, Isolated from Sheep in Pará, Brazil

**DOI:** 10.1128/genomeA.00208-16

**Published:** 2016-03-17

**Authors:** Jorianne T. C. Alves, Adonney A. O. Veras, Ana Lídia Q. Cavalcante, Pablo H. C. G. de Sá, Larissa M. Dias, Luis C. Guimarães, Eziquiel Morais, André G. M. Silva, Vasco Azevedo, Rommel T. J. Ramos, Artur Silva, Adriana R. Carneiro

**Affiliations:** a Federal University of Pará, Center of Genomics and System Biology, Laboratory of Genomic and Bioinformatics, Belém, Pará, Brazil; b Federal University of Pará, Campus of Castanhal, Pará, Brazil; c Institute of Biological Sciences, Federal University of Minas Gerais, Belo Horizonte, Minas Gerais, Brazil

## ERRATUM

Volume 4, no. 1, e01664-15, 2016. Page 1: The byline and affiliation line should read as given above.

